# Using Pupillometry to Assess the Atypical Pupillary Light Reflex and LC-NE System in ASD

**DOI:** 10.3390/bs8110108

**Published:** 2018-11-21

**Authors:** Georgina Lynch

**Affiliations:** Elson S. Floyd College of Medicine, Washington State University, Spokane, WA 99210-1495, USA; georgina.lynch@wsu.edu

**Keywords:** autism, biomarker, pupillary light reflex, locus coeruleus, eye tracking, screening

## Abstract

With recent advances in technology, there has been growing interest in use of eye-tracking and pupillometry to assess the visual pathway in autism spectrum disorder (ASD). Within emerging literature, an atypical pupillary light reflex (PLR) has been documented, holding potential for use as a clinical screening biomarker for ASD. This review outlines dominant theories of neuropathology associated with ASD and integrates underlying neuroscience associated with the atypical PLR through a reciprocal model of brainstem involvement and cortical underconnectivity. This review draws from animal models of ASD demonstrating disruption of cranial motor nuclei and brain imaging studies examining arousal and the influence of the locus coeruleus norepinephrine (LC-NE) system on the pupillary response. Pupillometry methods are explained in relation to existing data examining the PLR in ASD and pupillary parameters of constriction latency and tonic pupil diameter as key parameters for investigation. This focused review provides preliminary data toward future work developing pupillometry metrics and offers direction for studies aimed at rigorous study replication using pupillometry with the ASD population. Experimental conditions and testing protocol for capturing pupil parameters with this clinical population are discussed to promote clinical research and translational application.

## 1. Introduction

Since autism spectrum disorder (ASD) was first described by Leo Kanner, maladaptive behaviors impacting socialization and language continue to be the diagnostic indicators of this disorder across the lifespan [1]. Indicators include repetitive, restrictive patterns of behavior, lack of social reciprocity, and difficulty with social communication and language [2]. Recent data supports changes in brain function resulting from behavioral interventions influencing neural plasticity, and the developing visual pathway [3]. Levels of severity reflect distinct forms of neuropathology, making generalization of findings related to diagnostic markers and treatments difficult. Given the heterogeneity of presenting behavioral symptoms, diagnosis remains challenging, warranting the need for objective biomarkers. Methods capitalizing on assessment of the visual pathway as a mechanism for assessing brain function in ASD in a non-invasive manner hold promise for addressing challenges associated with ASD screening.

With advances in technology, pupillometry is a promising method for examining brain function and physical biomarkers within ASD. Given well established evidence describing plasticity of the developing brain [4], a review of work in neurodevelopment and dominant theories outlining core features of ASD provide insights into application of pupillometry to studies aimed at biomarker screening. Based on existing models of ASD neuropathology, recently documented animal models of ASD [5], and emerging data from clinical trials of pharmacotherapy [6], this review describes neuromodulation of cranial nerves subserving the pupillary light reflex (PLR), and associated primary process systems contributing to clinical symptoms of hyperarousal and anxiety found in the ASD population. Specific biometrics for assessing the PLR will be addressed. Despite controlling confounds such as ambient lighting and light stimulus duration and intensity, some degree of sensory perception takes place and visual processing in cortex occurs in response to changes in luminance as documented with eye-tracking paradigms [7]. Pupillary reactivity is influenced not only by light, but also by top-down processes in which perception effects pupillary change and activity in the PFC may influence regulation of the reflexive PLR [8]. Because pupil reactivity may not be reduced to the simple PLR, just as with other biometrics, PLR serves as a corollary of brain function, and may be influenced by other factors, including arousal related to the testing experience itself.

The intent of this review is to inform future experimental research using pupillometry and the application of these methods to analyzing specific pupillary light reflex (PLR) parameters holding promise for screening for ASD. Equally important is distinguishing the use of eye-tracking methods examining visual attention, from the specific PLR parameters captured using pupillometry. This review outlines underlying neural processes associated with ASD, examining data to support future studies aimed at earlier diagnosis based on the pupillary reflex test (PRT) and analysis of pupillary parameters showing sensitivity for differentiating typical development from ASD. The studies reviewed extend an established hypothesis of “under-connectivity” in the cortex to include a brainstem model of atypical PLR in ASD. 

Studies examining pupillary response in the ASD population lend insights into models integrating behavior with PLR biometrics, supporting future work aimed at refining objective clinical screening practices. This review provides background on specific pupillary parameters emerging as promising for impacting screening practices, including analysis of constriction latency and tonic pupil size. To address the limitations and subjective nature of current diagnostic approaches, translational application of PLR data may be used to evaluate the visual neural pathway in relation to tests of cranial nerve function used in a clinical neurological exam. A reciprocal model of underconnectivity between brainstem and cortex within ASD is explained in relation to these pupillometry methods to examine further the PLR as a potential screening approach. 

Established models of neuropathology of ASD frame emerging stratification of subtypes based on brain imaging and eye-tracking data. The relationship between subcortical structures and activation of primary process systems (sensory and motor) describe neural circuitry impacting cortical activity and expression of higher order social behaviors, focusing on the visual neural pathway. Pupillometry is a method for measuring arousal and describes neural activity occurring in the locus coeruleus-norepinephrine (LC-NE) system, providing a sensitive indicator of autonomic nervous system (ANS) function [9,10]. ANS regulation, mediated by neural circuits in the brainstem impacts acquisition of higher-order functions such as language, cognition, and socialization. Assessment of arousal via primary process systems supports a model of brainstem involvement as a contributing factor in the neuropathology of ASD. This model is paradigm-shifting, taking a concept of ASD as primarily based in behavioral disinhibition associated with neural activity in prefrontal cortex (PFC), to a reciprocal model of atypical PLR and physical symptoms associated with anxiety which are influenced by bottom-up processes and neuromodulation of cranial motor nuclei. It has been hypothesized that lack of connectivity between brain regions may contribute to maldevelopment of cortical processes in ASD [11,12]. The literature suggests this under-connectivity in cortex also impacts extensive reciprocal pathways modulated by structures in the brainstem, impacting language development during the early postnatal period when fundamental skills such as visual attending and social engagement with caregivers are acquired [13]. 

## 2. Models of Neuropathology-Functional & Anatomical Differences Underlying ASD

The literature supports distinct emerging subtypes predicting developmental outcomes, response to interventions, and degree of intellectual impairment and health complications [14,15]. Subtypes of ASD shift prior views and conceptualization of ASD from a disorder based on a continuum of severity to one of categorical differences with specifiers for comorbid medical conditions such as genetic disorders or seizures [2]. The search for the “broader autism phenotype” [16] and a subtype of autism characterized by neuroanatomical differences in brain development over the lifespan have emerged within the ASD literature [17,18,19,20]. These data support translational applications; however, stratification of ASD is still in its infancy. Developmental trajectory of milestone attainment, brain growth, and language outcomes have been analyzed for patterns of delayed, versus deviant, development to more specifically characterize ASD subtypes [21,22]. Physical characteristics associated with neuroanatomical structures in large clinical studies support emerging classification of endophenotypes associated with brain growth and ASD risk [23,24]. These models include precocious overgrowth of amygdala followed by plateau later in development and excess cerebral volume associated with head circumference and regression versus non-regression behavioral characteristics [25,26]. Longitudinal studies and prospective sibling studies have indicated distinct clinical subtypes, in which increased brain growth and retrospective analysis of head circumference in preschool boys predict ASD males with regression, predating behavioral symptomatology. These data suggest a distinctly different neuropathological process underlying a subtype of ASD with regression, and categorically different forms of the disorder observable early in development.

## 3. Underconnectivity & Inefficient Neural Processing

Well-documented studies using fMRI document differences in cortical processing of individuals with ASD in comparison to IQ-matched controls, and increased activation in occipital lobe and right hemisphere brain regions on tasks related to verbal memory [27]. Less functional brain connectivity has also been documented, which includes increased cerebral volume and altered white and gray matter primarily involving PFC [28]. Many studies highlight dysfunctional recruitment of higher-order cortical processes and a lack of connectivity between brain regions, influencing behavioral inhibition, auditory processing, and challenges with visual processing and social interaction [29,30,31]. It has been posited that underconnectivity between brain regions and abnormal development of pyramidal neurons in PFC results from atypical synaptogenesis, and a lack of pruning of overabundant neurons to create efficient neural pathways, implicated in the “under connectivity hypothesis” of ASD, describing micro- and macro-structural anomalies [32,33,34]. These findings demonstrate under-connectivity of PFC with other regions of the brain, reduced projections to subcortical structures, and atypical development of pyramidal neurons and dendritic spines, further exacerbating under-connectivity between cortical and subcortical regions. Disruption of cytoarchitecture impacting synaptogenesis during the earliest stages of cerebral development within ASD and overgrowth of neurons comprising the neocortex create more localized connectivity due to decreased projections of axons and excess connectivity in isolated regions of the brain [34]. Consequently, overabundance of neurons in PFC, and inefficient neural pathways pose extensive impact across brain regions. This underconnectivity and lack of neural pruning contribute to greater brain volume and to less precise cortical processing, but more importantly result in imbalance between excitatory and inhibitory neural activity. 

Theories associated with neuropathology of ASD describe deficient neural connectivity within and between brain regions and the influence of environmental stimuli on behavioral response mediated by subcortical circuitry [35,36]. An emerging body of research examining ASD and neural plasticity has documented changes in neural activity using EEG with behavioral changes associated with the efferent visual pathway in response to intensive visually based intervention [37,38]. Positive outcomes in behavior measured by joint attention and visual processing in infants and toddlers approximated normalized brain function in comparison to age-matched controls, suggesting delayed maturation of primary process systems which can be mediated by intervention [3]. 

## 4. Valproic Acid (VPA) Animal Model of ASD and the Visual Neural Pathway 

Decreased connectivity between cortical regions, hypothesized to be due in part to localized overconnectivity contributing to excitatory/inhibitory imbalance, has been documented in animal models of neurodevelopmental disorders [39,40,41]. A distinct clinical phenotype of ASD has recently been documented, linking a zebra fish model of disrupted development of the visual system to physical clinical features of ASD [42]. Perinatal conditions affecting the developing visual neural pathway have been implicated in infants later diagnosed with ASD, indicating a predisposed atypical maturational process related to visual attention [43]. This profile of development reflects disruption of neural systems affecting the efferent visual neural pathway modulated by cranial motor nuclei impacting eye gaze and pupil response. Disruption of cranial motor nuclei occurs at an interval just prior to neural tube closure influencing later development of primary process systems. These systems include auditory sensation, oral motor speech production, and visual attending, all impacted at varying degrees of severity within ASD. Effects of VPA exposure on development of cranial motor nuclei has been examined in rodents mirroring a model of brainstem lesion found in a clinical autopsy case of ASD. [44] VPA exposure resulted in malformation of motor nuclei of trigeminal, hypoglossal, and abducens, and when delayed to gestational day 12.5, the oculomotor nuclei were also significantly reduced with no observable physical anomalies or asymmetries visible after birth. This animal model of ASD describes deleterious effects on CNS development affecting cranial motor nuclei critical to primary process systems. The model has been replicated demonstrating deficient motor coordination and gait, lower sensitivity to pain, and an increase in locomotor/stereotyped behaviors, all common to the ASD endophenotype [45].

Following the VPA model, sodium valproate has been identified as a teratogen associated with disruption of neural tube closure, implicated in administration of antiepileptic medications during pregnancy [46]. Clinical studies reported significant risk for cognitive deficits and increased risk of ASD associated with prenatal exposure to VPA, prompting the US Federal Drug Administration to release a drug safety communication warning regarding its association with neurodevelopmental disorders [47]. Following the underconnectivity hypothesis and the VPA model, given extensive projections extending from the locus coeruleus (LC), disruption of cranial nuclei serves to provide a plausible substrate of underlying pathology in relation to the atypical PLR. 

## 5. Brainstem Circuits Influencing Maladaptive Behavior and Language Acquisition in ASD

Deficient neuromodulation at the primary process level impacting the thalamo-cortical system and excitatory/inhibitory neural activation in the cortex influences behavioral regulation, learning, and language development to varying degrees. Higher order cortical skills rely on fundamental pre-linguistic behaviors such as eye gaze, joint attention, visual attention to faces, and following gesture and symbolic language to promote language acquisition [48,49]. Given persistent challenges with sensory and motor skills across levels of severity, the behaviors associated with ASD implicate cranial motor nuclei subserving deficiencies in primary process systems. Deficient modulation of cranial nerves (CN II and CN III) affects the neural pathway of the visual system which must function efficiently for developing physical behaviors of joint attention and use of eye gaze for identifying dynamic changes in facial expression and nonverbal exchanges between communication partners. These eye gaze patterns have been shown to be deficient in ASD, impacting social interaction with caregivers [50,51,52]. Analysis of neuromodulation of cranial nerves affecting the visual neural pathway has not been reported heavily in the eye-tracking research literature related to ASD. Studies have emphasized visual attention and “visual preference”, rather than reporting measures of oculomotor utility and pupillary response directly modulated by the LC-NE system. 

Whereas the LC-NE system plays a significant role in arousal, it is also well documented that the amygdala is structurally and functionally deviant in ASD in comparison to typical development [53]. The influence of the amygdala on conditioned fear responding has been well characterized [54,55]. Individuals with ASD are more likely to show a heightened state of arousal and activation of amygdala in response to faces depicting emotional states when compared to typically developing populations [56,57]. Thus, the unfamiliar face serves as an unconditioned fear stimulus for individuals with ASD, resulting in hypervigilant amygdala activation, contributing to eye gaze aversion. Furthermore, individuals with autism demonstrate visual preference for objects over faces, suggesting a fear response to social stimuli [58]. The literature reveals a brainstem model of visual attending deficits in ASD describing heightened arousal and deficient modulation of cranial motor nuclei and the thalamo-amygdala pathway, which subserve deficiencies in higher order cortical processes. Consistent with performance by patients with lateral amygdala damage, individuals with ASD demonstrate eye gaze avoidance and hyperarousal responses to “social threat cues” and these responses appear to be automatic, with minimal cortical processing involved [59].

## 6. Eye-Tracking Methodology & Pupillometry to Measure Functional and Anatomical Differences

To examine hypervigilant responding in ASD, eye tracking methods directly measuring the pupil response record LC activity underlying observable behaviors and physical features of arousal. Reflexive responding to visual stimuli posing “threat cues” results from neurotransmission within the midbrain, emanating from the LC, triggering the “fight or flight” response. This response is the result of increased production of norepinephrine (NE), and preparing the body for responding by influencing cortical processes [59]. A two-part system coupling subcortical and cortical processing of visual stimuli expands the under-connectivity model beyond cortical localization and describes decreased connectivity of LC projections from subcortical systems throughout the cortex. This model emphasizes a state of hyperarousal documented in studies of ASD examining pupillary response, heart rate variability, resting sinus arrhythmia, and externalized behaviors associated with fear and anxiety, in which hyperphasic pupillary response is observed with blunted HRV reflecting allostatic load [60]. Eye-tracking studies with the ASD population have demonstrated increased visual attention for familiar faces over unfamiliar faces [61], aversion to direct eye gaze posed from human models on tasks related to social interaction [62], and eye gaze aversion on tasks related to imitation [63]. fMRI data incorporating eye tracking methods further supports eye gaze aversion in tandem with amygdala activation in response to faces, and to body posture with faces obscured, depicting “emotional body language” commensurate with affective responding to unfamiliar faces as “social threat cues” [64].

## 7. Pupillometry to Measure ANS Function in ASD & LC-NE Activity 

Assessment of pupillary response is a particularly sensitive measure of reflexive, autonomic responding mediated by neural activity generated within the LC [65,66]. Changes in pupil dilation result from the interaction of excitatory and inhibitory neural activity within the sympathetic and parasympathetic divisions of the ANS. In typical development, miosis (pupil constriction) occurs in the presence of an increase in luminance or ambient light, reflecting afferent and efferent neural pathways within the visual system and the activation of the parasympathetic nervous system in response to changes in lighting conditions [65]. Conversely, within the intact ANS, under dark adapted conditions, mydriasis (pupil dilation) occurs, reflecting adaptation to the environment, increasing the amount of light entering both pupils. These changes in diameter can be directly observed and measured, functioning as a protective mechanism for perception of objects and threats within the environment, and subsequently returning to homeostasis via the parasympathetic nervous system, providing continual adaptation to environmental changes. The pupil also reflects cognitive load, which can be influenced by arousal, demonstrating mydriasis during sustained processing of cognitive tasks; as task demand increases, pupil dilation also increases [67]. As a measure of neurologic infarct in clinical cases involving the mydriatic pupil (sustained dilation in response to a light stimulus), the symptomology suggests potential disease, trauma, or drug toxicity, and can indicate increased cranial pressure reflecting potential damage to or compression of, the third cranial nerve and dysregulation of neural activity in the brainstem [68,69].

**Figure 1:**
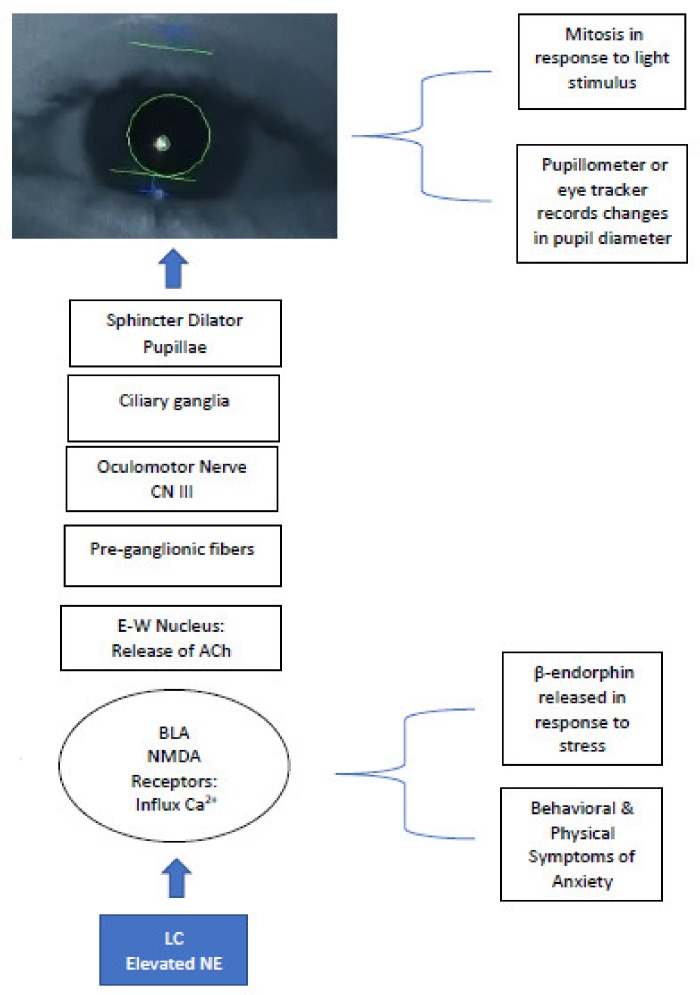
LC-NE system eliciting PLR and efferent visual neural pathway–modulation of NMDA receptors and cranial nerves produce pupillary change; elevated Ca^2+^ at NMDA receptors increase NE levels = hyperarousal/sympathetic state in ASD, inducing sustained mydriasis in response to light stimuli. LC = locus coeruleus; NE = norepinephrine; BLA = basolateral amygdala; NMDA = *N*-methyl-D-aspartate; E-W = Edinger Westphal; ACh = acetylcholine.

Given the sensitivity of pupil measurement as a proxy for LC-NE activity, pupillometry can be used efficiently with the ASD population as a less invasive measure of brain function and provides relative ease of administration in comparison to MRI and EEG. When used in tandem with fMRI, pupillometry offers a comprehensive view of the interaction between behavior and activation of neural pathways influenced by subcortical structures in the brainstem. The LC-NE system influences modulation of parasympathetic nerve fibers activating the PLR neural pathway, originating at the Edinger-Westphal nucleus and synapsing at the ciliary ganglion, controlling the pupillary sphincter, the muscle of the eye controlling constriction (Figure 1) [70]. 

The efferent visual pathway is typically assessed in a subjective manner within a clinical neurological exam. Current computer-based eye tracking systems can be used to objectively record and monitor change over time in relation to the study of modulation of cranial nerves, arousal, and visual motor performance. Emerging technology using hand-held equipment also holds promise for translational clinical research with healthcare providers, given the ease and simplicity of a handheld pupillometer which can capture PLR metrics quickly and easily [71]. Pupillometry can sensitively detect changes in the LC-NE system, by assessing integrity of the function of cranial nerves impacting pupil response. Eye-tracking and pupillometry has been used with the ASD population in studies examining eye gaze and social preferences [72,73,74,75]. When used to examine brain function in ASD, the use of eye-tracking technology to perform pupillometry with direct light stimuli as done with the PRT is particularly useful to investigate interactions between pupil reflex, heart rate, and other measures of autonomic nervous system function to directly measure firing of LC neurons in the absence of decision-making and attentional tasks. Using direct light stimulus presentation, the PRT sensitively detects parasympathetic response in a reflexive manner, whereas eye-gaze measurements using traditional eye-tracking methods are influenced by confounds impacting pupillary response reflective of cognitive processes and visual attention.

## 8. Pupil Response as a Useful Biomarker for Assessing Brain Function in ASD 

**Figure 2:**
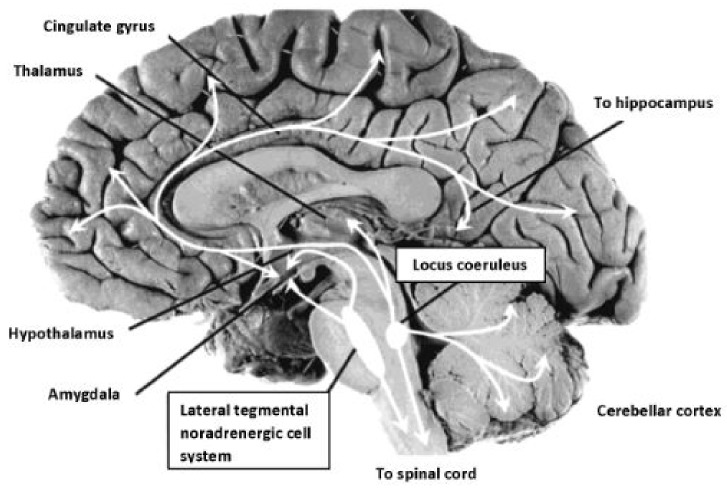
Noradrenergic projections from the locus coeruleus, site of synthesis of NE, modulate CN II & CNIII, controlling the PLR. LC neurons located in the pons project to subcortical structures associated with hyperarousal modulating neural activity and extend throughout cortex also directly effecting pupillary response to a light stimulus. Image reproduced from Strawn & Geracioti (2008). Photograph: Patricia Brown, Ph.D., University of Cincinnati. Public domain.

The PLR serves as one physiologic marker of ANS arousal emanating from the brainstem. Cranial nerves activate the pupil response via the optic nerve, CII (afferent) and the oculomotor nerve, CNIII (efferent), stimulated by release of acetylcholine (Ach) at the Edinger-Westphal nucleus, and modulated by the LC, a subcortical nucleus located bilaterally in the rostral dorsal pontine tegmentum [76]. The LC is the principle site for synthesis of NE, the neurotransmitter responsible for modulation of cranial nerves subserving primary process systems and activation of the sympathetic nervous system, observed in the form of increased heart rate, pupillary changes, and perspiration. The LC has been described extensively as it relates to neuromodulation of the autonomic nervous system [77,78]. The LC-NE system is implicated in sleep/wake regulation, stress response, and cognitive performance [79]. The LC-NE system involves inputs projecting from medial frontal cortex and the lateral hypothalamus, resulting in excitatory activity within the LC, generating significant levels of NE [80]. As illustrated in Figure 2, extensive efferent projections leave the LC from the brainstem, interfacing with diverse neural circuits throughout the cortex and spinal cord. 

The projections are extensive, affecting neural networks within and across forebrain documented through EEG measures [81]. Low levels of LC-NE activity have been documented in relation to low levels of arousal and alertness. High levels of LC-NE activity result in increased alertness and sympathetic response induced by the sudden appearance of environmentally salient stimuli. The relationship between LC-NE activity and arousal has been demonstrated in the acoustic startle reflex paradigm based on measurements of response to unconditioned stimuli and observed in cases of post-traumatic stress disorder [80]. As it relates to cognitive processes such as attention and decision-making, an inverse relationship between LC-NE activity and ANS response has been shown in which pupil diameter increases and is associated with optimal performance at an intermediate level of tonic activity, and attenuated phasic responses are associated with a decrease in performance. This pattern has also been demonstrated in relation to arousal and task performance, as posited by the adaptive gain theory describing the LC tonic and phasic modes in response to simple attentional tasks and task performance [81,82]. Increases in LC activity are associated with the outcome of task-related decisions coupled with behavioral responding and accuracy, thus costs and benefits of behaviors and rewards are strongly correlated with exploration of the environment via visual attention and pupillary changes. In the tonic mode, LC activity is observable, but distractibility increases, suggesting the LC-NE system influences decision-making, supporting the adaptive gain theory and the influence of NE on alertness and cortical processing.

Pupillometry has been shown to be a valid and reliable measure of LC activity in typical populations [66]. More recently, the utility of pupillometry as a sensitive psychophysiological marker of the LC-NE system has been examined, demonstrating the P3 event-related potential (ERP) and pupil diameter as predictable covariates of neuromodulation of the LC-NE system [83]. Task-relevant stimuli and antecedent events have been associated with strong P3 amplitude. It has been hypothesized that pupil diameter reflects the tonic and phasic aspects of LC-NE activity, demonstrated through pre-stimulus measures of baseline pupil diameter relating to task engagement following the adaptive gain model of LC-NE function [82,84]. 

## 9. Phasic Hyperarousal and the “ASD Advantage” in Visual Tracking

In line with heightened arousal, an “ASD advantage” in visual tasks has been documented, in which young children with ASD demonstrate a hyper-phasic LC-NE system, and persistent heightened arousal, which has been suggested to contribute to an increase in focused visual attention, outperforming typically developing two-year olds on visual search tasks [85]. This phasic state was documented in an increase in tonic pupil diameter measurements in which a pupil diameter increase of 2 mm was associated with a 66% increase in performance. As visual task difficulty increased, visual attention and pupil diameter increased in the ASD group, demonstrating heightened alertness observable through more frequent, sustained phasic states in comparison to controls. This model of hyper-phasic LC-NE activity is suggested to have an influence on performance for visual tasks and implicates a hyper-focused visual attentional state which within ASD drives repetitive behaviors and visually-based restricted interests. However, this model of hyperarousal is contrary to findings in typical populations in which increase in pupil dilation is associated with a peak in arousal just prior to anticipatory visual search related to goal directed outcomes. This has been demonstrated using EEG exemplified by the well-established Yerkes-Dodson effect in which there is an inverse relationship between tonic and phasic LC-NE activity and pupillary response [81].

Sensitivity of P3 amplitude and pupil measurements reflect increased performance and time on task measures in the presence of robust bursts of P3 activity and increased pupil diameter, indicating a pre-stimulus arousal state, indirectly reflecting LC-NE activity, and establishing pupil response as a proxy for firing of neurons in the LC [81]. Given extensive projections of neural pathways emanating from the LC to various regions of the brain, using pupil measurement to record physiologic changes reflecting arousal warrants further investigation in the ASD population. This methodology can help to better understand how the under-connectivity hypothesis may intersect with a theory of brain stem involvement and dysregulation within the LC-NE system, characterized by a persistent sympathetic state. 

## 10. Pupillary Response, Modulation of the LC-NE System, and Elevated Arousal in ASD

The LC-NE system modulates arousal through recruitment of amygdala activation and modulation of NMDA receptors. Elevated NE levels reflect failure of NMDA receptors to mediate influx of Ca^2+^ and modulate synaptic activity, which can result in an increase in endogenous opioids, such as ß-endorphin, producing increased tolerance to pain and an increase in repetitive behaviors, commonly observed in cases of ASD. The relationship between excess endogenous opioid production and increased NE may play a role in the elevated arousal states observed in ASD. This relationship has been examined in animal and clinical populations, demonstrating increases in behaviors characteristic of those found in ASD (self-injurious behavior, repetitive behavior, aggression) which reflect similar behaviors observed during opiate withdrawal, and can be reduced when opiate antagonists are administered [86]. Earlier studies examining low-dose pharmacotherapy using the opioid antagonist, naltrexone, yielded reports of increased social initiation and affective responses following administration in animals [87,88]. Improvements in prosocial behavior were documented in individuals with ASD when given very low doses of the drug [89,90]. Although these findings require further investigation, they hold promise for pharmacotherapy intervention targeting modulation of the sympathetic ANS, such as the use of the β-adrenergic antagonist propranolol, shown to reduce symptoms of anxiety and promote social engagement and cognitive performance in young people with ASD [91]. Studies examining the use of opiate antagonists and beta-blockers for ASD in animal models and clinical trials show promise in contrast to frequently prescribed SSRIs or antipsychotics [92]. Pharmacotherapy models examining treatment of ASD to mediate the effects of hyperarousal, and elevated levels of NE on the central nervous system, targeting NMDA have provided positive preliminary outcomes to mediate hyperarousal and promote social engagement [93]. Neurotransmitters influencing NMDA activity include NE, glutamate, glycine, and gamma-amino butyric acid (GABA). These neurotransmitters produce widespread effects on neural transmission from brainstem throughout cortex. Glutamate and glycine bind to NMDA receptors, influencing uptake of Na^+^, K^+^, and Ca^2+^ ions, opening receptor channels, impacting excitatory and inhibitory synaptic activity, affecting alleostasis. Targeting the LC-NE system through pharmacotherapy has the potential to influence eye gaze motor control and social avoidance in ASD and to decrease social aversive and repetitive behaviors observed, when it is considered to enhance neuromodulation of NMDA receptors. Moreover, when left untreated, chronic excitatory NDMA activity has been implicated in neurodegeneration [94], aligning with physical and behavioral characteristics observed in the regressive subtype of ASD, and in later adolescence when adaptive skills begin to decline. The use of propranolol is also frequently prescribed to address hyperarousal and social anxiety. Given that the atypical pupillary response co-occurs with respiratory sinus arrhythmia and externalizing behaviors, propranolol has had positive outcomes for addressing these physical indicators of anxiety associated with ASD and for improving functional neural connectivity during cognitive tasks [95,96,97].

## 11. The Atypical PLR Observed in ASD—Valid Measures of the PLR

**Figure 3:**
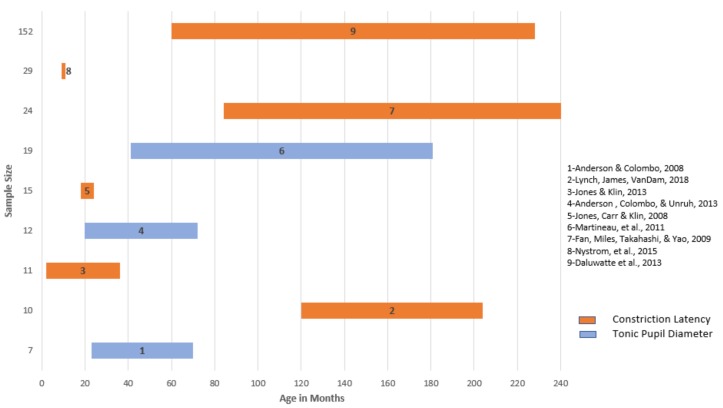
Eye-tracking studies examining pupillary parameters constriction latency and tonic pupil diameter by age and sample size, reporting significance for differences in comparison to typical development.

Observation of significant differences in the PLR have been shown in the ASD population consistent with a hyper- aroused ANS reflective of delayed constriction latency, deficient functioning of parasympathetic response to changes in lighting conditions, and excess production of NE [70]. With emerging technology in recent years allowing advanced computing techniques to capture precise pupil parameters, the body of experimental research targeting specific puillary parameters in ASD is limited, but promising, for developing pupillary metrics in comparison to typical development. Figure 3 outlines studies using eye-tracking technology across ASD samples measuring specific pupillary parameters first developed by Fan, Miles, Takahashi, & Yao [98], including constriction latency and tonic pupil size, consistently implicated in PLR measures of ANS dysregulation. Consistent across these studies is the demonstration of heightened sympathetic state in response to a light stimulus, although baseline measures and latency to constriction varied, the average latency to constriction for the ASD groups across ages was between 2000 and 3000 ms, indicative of a clinically “sluggish pupil” in samples ranging from 24 months to 20 years of age. Recently the reverse effect has been reported in one sample of a very young population of at-risk infants later diagnosed with ASD, in which the constriction latency was enhanced in comparison to typically developing infants, not diminished as other studies have demonstrated [99]. Limitations of those findings included a small sample and analysis of pupil diameter at maximal constriction, not latency, previously shown not to vary substantially between groups. However, a dysregulated ANS was observed, in line with other PLR studies. The clinically predictive value of that pupillary parameter for later diagnosis requires further investigation in relation to constriction latency and tonic pupil size. 

Eye-tracking paradigms examining the PLR in light-adapted and dark-adapted conditions have been used to analyze differences in baseline tonic pupil size and to study interactions between HRV [100], skin conductance (SC), and salivary alpha-amylase (sAA) and cortisol levels [101]. Tonic pupil size in children with ASD positively predicted group membership (ASD = 71%) in comparison to mental- and age-matched controls pupil diameter [102]. Within ASD, pupil diameter measures were found to be insignificant for mental age or level of intellectual impairment, suggesting the PLR was a sensitive measure for differentiating ASD from typical development. Following this eye-tracking paradigm, the pupil response was isolated as a reflexive behavior, demonstrating physiologic changes in response to light stimuli and illuminance levels, removing cognitive load confounds, thus measuring subcortical activity and activation of the LC-NE system. Fan, Miles, Takahashi, and Yao [98] measured transient PLR, utilizing the pupil reflex test (PRT), a simple measure of ANS response to presentation of a light stimulus in light- and dark-adapted conditions. Their results indicated significantly prolonged constriction latency, lower constriction velocity, smaller relative constriction, and uniform contraction anisocoria in the children with ASD in comparison to controls. These results were replicated in an older sample of adolescents, indicating atypical maturation of the visual pathway later in a stage of development at which primary process systems typically function most efficiently [103]. These data demonstrate decreased responsiveness of the parasympathetic nervous system, suggesting possible degenerative processes, demyelination, or dysregulation of the ANS. Overproduction of NE in the brainstem is also a plausible factor, resulting from deficient modulation of the cranial nerves necessary for efficient muscular control of the pupillary reflex (CNII & CN III), in tandem with increased arousal, suggesting the presence of excessive endogenous opioids, such as β-endorphin, which inhibit neurotransmission of NE [104].

Although greater pupil diameter and increased constriction latency appear to be generally consistent across studies, one study with older children documented results in contrast to decreased responsiveness of the ANS [105]. Results indicated smaller constriction size in the ASD sample in response to visual stimuli, predicting ASD group membership for 89% of participants. Although contrary findings were reported, results concurred with dysregulation of the ANS as cited in other PLR studies. Procedures varied from other methods and outcome measures and were based on presentation of socially relevant visual stimuli depicting faces, as opposed to a direct, single light stimulus typically used to assess pupillary changes. Given that cognitive demands of viewing socially salient stimuli influences change in pupil size, this factor could affect the outcomes. The differences in ambient lighting and environmental context also may have influenced pupil size given the interstimulus slides and luminance level changes emitted from the monitor. The contrary findings represent the importance of controlled experimental pupillometry methods using consistent ambient lighting conditions and light stimuli presentation for study replication to directly assess LC-NE function. For a more complete examination of PLR and ASD by pupillary parameter, methodology, and outcome, see studies cited in Supplementary S1.

## 12. PLR and Corollaries of ANS Modulation within ASD

Anderson, Columbo, & Unruh [101] investigated PLR to sensitively predict ASD group membership in children between the ages of 20 months and 6 years, analyzing interactions between pupil dilation, and salivary alpha amylase (sAA) (a putative correlate of NE), to measure the parasympathetic system in response to light stimuli. Co-variate measures of cortisol were analyzed to determine the influence of testing environment on arousal, and to determine if NE levels were indicative of pupillary response to the light stimulus, supporting sensitivity for use of pupil measurements in detecting subtle changes in ANS function. Consistent with other studies, tonic measures of pupil size significantly differentiated ASD participants from those with another developmental delay or typical development, in addition to sAA levels, and there was no significant difference in cortisol level. Results indicate sensitivity of the pupil response to presentation of light stimuli yielding a reflexive pupil response directly measuring integrity of ANS function via the LC-NE system.

Daluwatte et al. [100] conducted one of the first comprehensive studies in a large sample size examining PLR and heart rate variability (HRV) in ASD and other developmental disorders, citing results for school age children with ASD commensurate with greater pupil constriction latency and less relative constriction in response to the PRT. An inverse relationship between pupil response and HRV was reported, indicating increased resting heart rate, and decreased HRV in ASD post-stimulus presentation in comparison to typical controls, suggesting dysregulation of the ANS. These data demonstrated efficient return to baseline diameter (homeostasis) in typically developing participants in response to light stimuli, and reduced activation of parasympathetic activity in the ASD group. Resting HRV was not significantly different between ASD and other developmental delays, supporting the PLR as a sensitive measure differentiating ASD from typical development, holding potential for specificity when considering the return to baseline pupillary metric paired with constriction latency as a measure of homeostasis. ANS responding in the ASD group was not modulated in response to a light stimulus, as was observed in the other two groups. Furthermore, these results were not significant for IQ or level of intellectual impairment, indicating deficient functioning of ANS activity across cognitive level, and across levels of ASD severity. 

Given the extensive data set and robust sample size, Daluwatte et al.’s [100] study is one of the first to attempt to stratify the ASD sample according to chronological age, cognitive level, and use/non-use of medications affecting ANS response, in relation to physiologic indicators of ANS function assessed using PLR. This model for capturing PLR measurements in relation to cognitive and behavioral presentation supports sound research design aimed at examination of the PLR for noninvasive clinical screening of the developing visual pathway in neurodevelopmental disorders. It is likely that variability of results for studies using eye-tracking methodology to perform pupillometry is related to heterogeneity of the samples, inconsistent ambient lighting conditions, and to a lack of subtyping the behavioral profile. 

## 13. PLR as an Index of Maturation of the Visual Pathway

It is noteworthy that eye tracking data reveal the visual system appears to be intact at birth in infants later diagnosed with ASD but found to decline as the visual system matures [43,50]. Longitudinal prospective examination of infants over the course of early development beginning at 2 months of age indicate no significant differences from typical development on measures of preferential attention to the eyes at 2 months, with significant changes in mean measures of eye gaze emerging between 18 and 24 months of age. Typically developing infants demonstrated a sharp decline in attention to objects within the first year of life, reaching plateau between 18 and 24 months, and increasing attention to eyes and the human body following plateau. Those diagnosed with ASD by 36 months demonstrated significant decline in attention to eyes and body, and increased fixation on objects after baseline measures which had been commensurate with typical development for the first two months of life. Results suggest aberrant development of the visual system in the early postnatal period.

Jones and Klin’s [43] data suggest a potential degenerative neural process involving the visual pathway, and perhaps one in which intensive motor-based intervention might mediate deficits in the visual system as efferent pathways are more actively engaged during early development. Addressing brain plasticity, it is plausible that intensive environmental exposure to visual stimulation could promote experiential synaptogenesis, further strengthening the visual neural system if started very early in infancy, as demonstrated in EEG with changes in visual attention [3]. It is also likely the atypical PLR latency, in comparison to eye gaze measures alone, could prove correlated to greater degrees of subcortical involvement, and could be used for discriminating reflexive arousal associated with the ANS (i.e., PRT), from differences in visual attention and social preference (i.e., eye-tracking methods). 

Assessment of the PLR holds promise as a potential screening tool aimed at identifying children with ASD, in which maturation of the visual pathway shows decline or plateau on PLR measures, indicating atypical neurodevelopment in the context of co-occurring behavioral characteristics. The studies examined here support the PLR as a simple, reflexive physical mechanism by which ASD can be differentiated from typical development. The contrast between factors influencing oculomotor utility and visual preference has yet to be completely worked out in research design using current forms of eye-tracking technology and pupillometry. However, the use of PLR parameters such as constriction latency, tonic pupil, and dilation amplitude have begun to produce consistent findings in relation to sensitivity for typical vs. atypical responding and discriminating ASD from typical development across developmental levels. These PLR parameters are proving to provide reliable, valid outcome measures for pursuing the use of PLR in screening. Moreover, as technology advances, pupillometry serves to offer feasible methods for identifying atypical physical features of maturation as first line screening using a simple, non-invasive approach. Measurement of PLR is taken by health care providers at well-child checks, which aligns with an expedient and efficient way to objectively assess basic features of neural development and reliably identify atypicalities in the context of clinical evaluation in comparison to more involved procedures, such as EEG. 

## 14. Discussion and Future Directions

As further biometric studies examine the atypical PLR in ASD, the current body of literature is generally limited by inconsistencies in research design for capturing specific pupillary parameters. Attention should be paid for consistency in use of eye-tracking equipment, pupillometry procedures (i.e., ambient lighting conditions, luminance levels, light stimulus presentation), and behavioral protocols for study replication. This review highlights studies demonstrating consistent findings through study replication based on specific PLR parameters (constriction latency and tonic pupil) emerging within ASD research as viable for future inquiry. Because the pupillary system reflects sensory processing in addition to arousal, carefully controlled conditions are necessary to induce the PLR with consistency if these metrics are to hold promise for clinical screening. Results of PLR studies using eye-tracking methodology and visual stimulus paradigms limit interpretation of findings in relation to LC-NE activity. Although informative of other aspects of arousal and visual attention, these paradigms should not be considered in comparison to pupillometry using direct examination of the PLR when evaluating biometrics. In this regard, close attention should be paid to methods using direct light stimuli to assess the PLR versus presentation of inter-stimulus slides integrated with visual stimuli. Future research should examine translational application of the PLR and the relationship between modulation of cranial motor nuclei and the visual pathway. Findings obtained using direct light stimulus methods will serve to better inform future medical practice and help elucidate underlying neural circuits involved in ASD for potential testing of pharmacotherapy influencing the LC-NE system. As reviewed, studies using pupillometry with ASD populations have reported a broad range of developmental levels related to chronological age, language level, and intellectual ability. Stratification within study samples is still needed in research design to extend these preliminary PLR measures to translational application. Currently, the use of PLR as a screening tool holds promise for sensitivity, however, robust findings are still limited as to whether it would support differential diagnosis of ASD subtype or whether this biometric demonstrates specificity for identifying ASD in relation to other developmental disorders. Studies examining interaction effects of behavioral tools to characterize subtype of ASD in relation to biometrics of the PLR based on the data presented warrant further investigation to parse out the heterogeneity of the disorder and support early identification. 

## Supplementary Materials

The following are available online at http://www.mdpi.com/2076-328X/8/11/108/s1, Table S1: PLR studies by sample size, PLR measures, and main effects.
