# Supplementary material for: Using Pupillometry to Assess the Atypical Pupillary Light Reflex and LC-NE System in ASD
Source: Behav Sci (Basel). 2018 Nov 21;8(11):108. doi: 10.3390/bs8110108 (PMC6262612; doi:10.3390/bs8110108)
Supplement: Supplementary file 1 [file behavsci-08-00108-s001.zip › Table 1 PLR lit review behavsci-372881.docx]

| **Reference** | **Clinical**  **Group**  HR=High Risk | **Comparison group**  LR=Low Risk | **Stimuli**  ISI=  interstimulus interval | **Pupillary Measures/**  **Outcome Measures** | **Results, group comparisons for latency measures:**  **< means *faster*; > means *slower*; = represents no significant difference** | **Means/SD**  **Main effects**  *significant at *p* < .05  **significant at p <.001 | **Possible Explanation** |
| --- | --- | --- | --- | --- | --- | --- | --- |
| Nystrom, et al., 2018 | HR-ASD  Age: 9mos.  N=29, 7 F  Age at follow-up: 36mos. | HR- No ASD  Age:9 mos.  N=118, 62 F  TD  Age: 9mos.  N=40, 20 F  Age at follow- up: 36mos. | Computer monitor- Black ISI slides= 0.9 *lux*; white light flash 190 *lux*  16 slides with ISI video clips | PLR:  1.relative constriction  2.PLR latency | 1.HR-ASD < TD  HR-ASD < HRNoASD  HR-No ASD =TD  2.HR-ASD = TD  HR-ASD = HR-NoASD  HR-NoASD =TD | Significant main effects for Group (HR-ASD)  Scaled PLR values = 0-1  1. *F*(2,184) = 6.4, *p* = 0.002, ηp² = 0.065.  TD: *M* = 1.00, *SD* = 0.21  HR-ASD: *M* = 1.20, *SD* = 0.24  HR-NoASD:*M* = 1.06, *SD* = 0.23  2.No significant difference | “…an atypical PLR is more likely to index general atypicalities in brain development than selective disruptions of the so-called ‘social brain’…”  “…finding suggests atypicalities in sensory processing play an important role in the early development of ASD.”  PLR latency relates to “elevated risk” for those not diagnosed at 36 months  Limitation: 2 sites using non-identical methodology & normalized scales for PLR measures |
| Naples, et al., 2018 | ASD  Age: 4.42-11.3yrs  *M* age=7.77  N=25 (5 F) | TD  Age: 6.59  N=26 (9 F) | PLR: Computer monitor: black slide and white flash: 133ms  EEG: Resting data recorded while participants watched a video on a computer screen | PLR:  1.relative constriction  2.latency to constriction  3.constriction velocity  EEG &Clinical Behavioral Tools | 1.ASD=TD  2.ASD>TD*  3.ASD<TD* | 1.PLR constriction correlated with:  SRS Subscale Cognition  (*r* = .459, *p =* .003)  2. PLR latency correlated with:  a. ADOS severity score  (*r=*.359; *p*=.093)    b. Vineland Socialization Domain:  (*r =* .586*, p*= .005) | “the relationship among the PLR and ASD symptomology suggests increased noradrenergic activity, indicated by increased PLR latency and attenuated constriction”  Prolonged PLR latency correlated with degree of severity of ASD; correlations between physical measure of arousal (PLR) and measures of behavior may support subtyping ASD |
| Lynch et al., 2018 | ASD  Age:13.11-17.2 yrs  *M*=16.15  N=10 (2 F) | TD  Age=11.5-17.8yrs  *M*=15.20  N=12 (5 F) | Binocular infrared;  Direct light stimulus;  1.0 *lux* ambient conditions  Dark-adapted  conditions | PLR:  1.constriction latency (t_L_)  2.Return to baseline (t_R_) pupil diameter  3. combined (t_L_) X (t_R_) | 1. ASD > TD  2. ASD > TD  3. ASD > TD | 1. ASD >TD*  ASD: *M =*3670 ms; *SD =*2360 ms  TD: *M =*2740 ms; *SD* =1200 ms  2.ASD > TD*  ASD: *M* = 4610 ms; *SD* =2080 ms  TD: *M* = 2580 ms; *SD* =1930 ms  3. Combined (t_L_)X (t_R_) discriminated ASD from TD* | Prolonged latency “…suggest contribution of a “bottom-up” process affecting arousal and physical responding influenced by neural activity within the LC, impacting efficiency of the pupillary reflex.”  Supports reflexive responding of the pupil to discriminate ASD from TD  Dysregulation of homeostasis |
| Dinalankara, et al., 2017 | ASD  Age:2-6 yrs N=60 | TD  Age: 2-6 yrs N=57 | Computer monitor-white light automated flash; 5.8 *lux* ambient lighting  Initial video clip | PLR:  1.Base pupil radius (mm)  2.PLR latency (ms)  3.Constriction time (ms)  4.Constriction (%)  ANS Deficits- parent report | 1.ASD =TD  2.ASD >TD*  3. ASD =TD  4. ASD =TD | Significant main effect for age (ASD); Mean/SD:  1. ASD:3.26mm/0.37  TD: 3.24mm/0.42  2. ASD:246.17/18.20*  TD: 235.77/16.01  3. ASD: 382.56/59.00  TD: 372.15/48.05  4. ASD: 19.01/7.47  TD: 19.69/6.55  Diminished base pupil radius & PLR latency beginning at~ 24 months | Prolonged PLR latency in ASD differentiated TD and ASD  ASD group demonstrated more ANS dysfunction correlated with PLR parameters |
| DiCriscio & Troiani, 2017 | ASD  Age: 5-16 yrs *M*=8.95  N=42, (21 F) | None | PLR:  Computer monitor: black ISI slides and white flashes; *sustained* stimulus presentation | PLR:  1.amplitude-dilation (A_D_)  2.amplitude-constriction (A_C_)  3. latency to max constriction (t_CL_)  4. latency to max dilation (t_DL_)  Light vs. Dark Adapted  Gender  SRS score | ASD/Condition:  1. A_D_ dark > A_D_ light  2. A_c_ light > A_c_ dark  3. t_CL_ light > t_CL_ dark  4. t_DL_ light = t_DL_ dark | SRS T-score/severity correlated with:  A_D_ = (r=-0.55, *p*<0.001)  A_C=_ (r = −0.40, *p* = 0.008)  A_DL=_ (r = −0.55, *p <*0.001)  t_CL=_(r = −0.38, *p* = 0.01)  Significant main effect for:  Condition  (F(4,77) = 142.87, *p* <0.001*)*  No effect for gender  A_D_ differentiated ASD based on  SRS T-score** | “… amplitude of pupil dilation in the current paradigm was found to be a significant predictor of autism features.”  “…it is plausible that certain physiological pupil responses within task-based paradigms are linked to one domain of autism traits…others are associated with baseline pupil diameter and/or automatic, reflexive responses.” |
| Nystrom et al., 2015 | HR-ASD  Age:10 mos.  N=29, 16 F | LR- ASD Age=10 mos. N=15, 7 F | Computer monitor-  Black ISI  slides= 0.9 *lux*; white light flash 190 *lux*  16 slides with ISI video clips | PLR:  1. relative constriction  2. latency to constriction onset  3. tonic pupil size | 1. HR-ASD > LR-ASD*  2.HR-ASD < LR ASD  3. HR ASD = LR ASD | Significant main effect for Group (HR-ASD)  1.HR-ASD*: *M*= 47.84%; *SD*= 5.84%  LR-ASD: *M* =39.89%, *SD* = 8.29%  2.HR-ASD*:  *M*= 261.38 ms, *SD* 19.27 ms    LR-ASD:  *M*= 276.00 ms, *SD*= 20.94 ms  3. HR-ASD:  *M*= 4.80 mm, *SD*= 0.56 mm    LR-ASD:  *M*=4.68 mm, *SD* = 0.54 mm | “…supports theories emphasizing sensory abnormalities as well as the inclusion of sensory hypo- or hyper- reactivity in the diagnostic criteria for autism”  Early cholinergic disruptions affect the hypersensitive PLR in high-risk ASD |
| Nuske et al., 2014 | ASD  Age: *M* =4.02 yrs N=25 (4 F) | TD  Age: *M*=4.27 yrs  N=21 (3 F) | Grey ISI slides; scrambled images | PLR:  Resting state pupil diameter  Mullen Scale Score | No group differences | ASD: *M=*3.57, *SD= 0.35*  TD: *M*=3.60, *SD*=0.41  *t*(44) = 0.17, *p* =0.86  Resting state pupil diameter not correlated with Mullen score  (*p* range = 0.17-.92) | “Contrary to expectations, no atypicalities in tonic pupil size were identified.”  Resting state pupil physiology may not be as strong a predictor for ASD as PLR; resting state hyperarousal not associated with tonic pupil size |
| Jones & Klin, 2013 | ASD  Age: 2 to 6 mos. N=11 (0 F)  Participants tested at ten different time points between 2-24mos. of age | TD  Age: 2-6mos. N=25 (0 F)  Participants were tested at ten different time points between 2-24 mos. of age | Binocular infrared  Video scenes containing a woman playing role of caregiver playing childhood games | Visual fixation & saccades  Developmental change in fixation to eyes, body, object, mouth | Decline in fixation by infant  ASD < TD* in visual fixation to the eyes, body, and objects  Visual fixation in ASD = TD at 2 mos., but declined over time by 24 mos. with ASD | Significant interaction of age by diagnosis between 2-24 mos.:  Eyes*: F_1,34_ =11.90, P =0.002  Body*:  F_1,34_ = 10.60, P =0.003  Object*:  F_1,34_ = 12.08, P =0.002  Not significant for age and diagnosis:  Mouth:  F_1,34_ = 0.002, P =0.965 | “…derailment of skills that would otherwise guide typical socialization, and this early divergence …suggests a means by which diverse genetic liabilities are instantiated, developmentally, into a spectrum of affectedness.”  Differences observed between 2-6 months of age |
| Anderson et al., 2013 (Study 1) | ASD  Age: 20-72 mos. *M*= 50.25 mos. N=12 1( F)  Down Syndrome (DS)  Age: 20 to 72mos.  *M*=48.67 mos.  N=9 (2 F) | TD  Age:20-72 mos.  M=51.73 mos.  N=11(1F) | Passive viewing on a computer screen  Grey slides; 3- minute viewing; 3.0 *lux* ambient conditions | PLR:  1.tonic pupil size  2.salivary alpha -amylase (sAA)-afternoon levels | 1.ASD > TD for pupil size*  2.ASD<TD for sAA*  Discriminant Analysis:  Tonic pupil size & sAA predicted ASD | Significant interactions:  All measures were *p* < .05.  Diagnosis/tonic pupil:  F(2, 25) = 6.244, p = .006, η^2^ = .333  ASD pupil size *M_adj_ =* 5.59mm  DS pupil size *M_adj_*  = 4.19mm  TD pupil size *M_adj_* = 4.53mm  Discriminant Analysis Correlations:  sAA: .745  Tonic pupil size: -.536 | Resting state hyperarousal in ASD is associated with increased pupil diameter and afternoon sAA levels.  A dysregulated ANS measured with PLR and ANS corollary sAA differentiates ASD from TD |
| Anderson et al., 2013 (Study 2) | ASD  Age: 39-73 mos. *M*= 57.78 mos.  N=18 (0 F) | TD (age range=33 to 79 months, mean age=52.26 months old, n=19, 0 F) | Viewing a computer screen that varied in brightness levels | PLR:  1.tonic pupil size  2.salivary alpha-amylase (sAA)-diurnal variation | 1. ASD >TD*  2.ASD < TD* | Significant interactions:  All measures were *p* < .05.  Diagnosis/tonic pupil:  F (1, 34) = 31.112, p > .001, η^2^ = .478  ASD pupil size M_adj_ = 5.573 mm  TD pupil size M_adj_ = 4.304 mm  Diagnosis/afternoon sAA:  Discriminant Analysis:  Λ = .529, χ^2^ (2, N = 49) = 29.269, p < .001  Correct classification ASD: 80.8%  Correct classification TD: 91.3%  False positive rate ASD: 19.2%  False negative rate ASD: 8.7% | “…replication of a dysregulated tonic pupil size and altered sAA in ASD implicates their potential as biomarkers for early identification.”  “…both measures have the potential to provide information about the neurological development in ASD and could provide potential targets for pharmacological intervention.” |
| Martineau et al., 2011 | ASD  Age:  41 to 181 mos.  *M* = 118 mos.  Mental age (MA):  35 to 136 mos. N=19 (3 F) | CA-matched  *M*= 116 months  N=19 (8 F)  MA-matched  *M=* 87 months  N= 19 (7 F) | Static color photos (neutral faces, virtual faces, objects); PLR measured using ISI blank black slides with ISI slides of static photos | Baseline pupil diameter | 1. ASD<MA  2. ASD < CA | Significant difference for ASD**:  ASD: M = 4.11 mm  (SEM) = 0.12  MA: M = 5.35 mm  (SEM) = 0.10  CA: M = 5.46 mm  (SEM) = 0.16 | “…participants with autism showed no initial differences in pupil diameter from the control, but did show a smaller constriction amplitude and slower constriction velocity than children with normal development”  “indicated a low level of ANS functioning. |
